# Evaluation of sexual function and sexual quality of life in women during the COVID-19 Pandemic: the Turkish case

**DOI:** 10.4314/ahs.v23i1.37

**Published:** 2023-03

**Authors:** Rojjin Mamuk, Sultan Yurtsever Çelik, Emine Temizkan Sekizler

**Affiliations:** 1 Nursing Department, Faculty of Health Sciences, Eastern Mediterranean University. Famagusta, North Cyprus; 2 Wound care unit of Bağcılar Training and Research Hospital of University of Health Sciences, İstanbul, Turkey

**Keywords:** COVID-19, quality of sex life, sexuality, sexual function, women

## Abstract

**Background:**

Sexual health is an important component of general health.

**Objective:**

To evaluate sexual function and sexual quality of life (SQOL) in women during the COVID-19 pandemic.

**Methods:**

This descriptive, cross-sectional study was conducted in Turkey. Data were collected via a Visual Analog Scale (VAS), Female Sexual Function Index (FSFI), and Sexual Quality of Life-Female (SQOL-F) questionnaire.

**Results:**

The mean FSFI score was 26.91±5.62, and 39.1% of the women had an FSFI score of 26.55 or lower. The mean SQOL-F score was 79.08±20.90. FSFI score was significantly associated with employment status (β=-0.661), partner education (β=1.698), sexual compatibility between partners (β=0.518), sexual satisfaction (β=0.230), fatigue level (β= -0.120), and frequency of sexual intercourse (β=0.160). In addition, SQOL-F score was significantly associated with sexual desire (β=2.625), satisfaction (β=1.338), pain or discomfort (β=1.274), age (β= -0.356), sexual compatibility between partners (β=1.984), and fatigue level (β=-0.981) (p<0.05).

**Conclusion:**

Less than half of the women participating in this study had sexual dysfunction, and overall SQOL was moderate to high. These results were associated with some descriptive characteristics of the women and were similar to those reported in pre-pandemic studies conducted in Turkey.

## Introduction

Over the course of history, humanity has faced numerous epidemics and mass deaths. Since the first reported case of novel coronavirus disease 2019 (COVID-19), millions of people worldwide have died^1^. In addition to its adverse impact on physical health and high mortality rate, COVID-19 also has negative effects on mental health^2^,^3^. Psychological problems that have been associated with COVID-19 include panic disorder, sleep disorders, anxiety, increased depressive symptoms, decreased positivity, and reduced quality of life^4^–^8^.

Sexual health is an important component of general health, and mass disasters affect reproductive health, especially in women^9^,^10^. Pandemics caused by infectious diseases are one of these mass disasters^11^. Therefore, people may experience difficulties meeting their sexual needs during the COVID-19 pandemic^12^. Levels of sexual satisfaction are associated with mental health and quality of life, and reduced sexual activity increases the likelihood of depression^13^,^14^. Several researchers have reported that the COVID-19 pandemic main directly cause sexual dysfunction (SD), lower sexual quality of life (SQOL), and a decrease in sexual desire^15^–^19^. In addition, the unfavourable conditions created by the pandemic affect communication between couples, sometimes leading to conflict. Conflictual relationships cause a decrease in compatibility between partners and in sexual activity^20^. However, some publications have shown no significant difference in people's sexual lives before and after the pandemic^21^. Although other studies have evaluated the sexual function (SF) of women in Turkey during the COVID-19 pandemic, there has been no large study evaluating women's SF and SQOL together^18^,^22^,^23^. Therefore, this study aimed to evaluate SF and SQOL in women living in Turkey and their associated factors by answering the following research questions:

(1) How is women's SF during the COVID-19 pandemic?

(2) What factors are associated with women's SF during the COVID-19 pandemic?

(3) How is women's SQOL during the COVID-19 pandemic?

(4) What factors are associated with women's SQOL during the COVID-19 pandemic?

## Methods

### Study type and sample

After obtaining ethical approval, a descriptive/relational cross-sectional study was carried out between April 10, 2021 and May 10, 2021. The sample size for the study was calculated based on the expected prevalence in the population. A recent meta-analysis conducted in Turkey was used as a reference for statistical power analysis^24^. The minimum number of participants required to estimate a prevalence of 39.6% with precision of 5% and confidence level of 95% was determined as 368. To reach the desired sample, 965 women were invited to complete an online questionnaire, and all of these women constituted the study population. A total of 728 of the 965 invited women responded. Of these, we excluded 40 women's responses because they did not meet the sampling criteria and 44 responses that included unrealistic answers (e.g., number of births >25, monthly frequency of sexual intercourse >85). As a result, the study was completed with 644 participants. The sampling criteria were being female, aged 18-50 years, being married or having a regular sexual partner, having no diagnosed SD, not being pregnant, puerperal, or menopausal, having no known mental or physical chronic disease, not having a current COVID-19 infection, and volunteering to participate in the study.

### Data collection process and tools

This study was conducted online during partial closures imposed because of the COVID-19 pandemic. For this reason, data were collected using an online questionnaire hosted on Google Forms. A link to the questionnaire was sent to the participants' personal social media accounts (e.g., Facebook, Instagram, Twitter), WhatsApp, and e-mail addresses. After obtaining permission from the group administrators of social media, WhatsApp, and similar groups with large membership, we informed the members about the study and invited them to participate in the online survey. Questionnaires created on the Google Forms platform can be configured tallow each participant to complete the survey only once and require all questions to be answered. These settings were used to prevent multiple form submissions from the same participant and potential data loss due incomplete submissions. The data collection tools used in the questionnaire included a sociodemographic information form, the Visual Analog Scale (VAS), Female Sexual Function Index (FSFI), and Sexual Quality of Life-Female (SQOL-F) questionnaire.

### Sociodemographic information form

We developed this form in light of the literature to determine the women's sociodemographic characteristics and various factors believed to potentially affect SF and SQOL^24^,^25^. The form included 20 questions including the women's age, education level, employment status, chronic disease history, partner's age and education, presence of SD in self or partner, family structure, income level, age at marriage, marriage duration, numbers of pregnancies, births, and children, and COVID-19 history.

### Visual analog scale (VAS)

The VAS is a unidimensional scale developed for pain assessment and is also used to determine individuals’ thoughts and feelings about various situations^26^. In the scale, individuals are asked to rate their opinion on a subjective situation between 0 (not at all) and 10 (very much)^27^. For this study, a numerical VAS was prepared in Google Forms and participants were asked to rate their compatibility with their partner, level of sexual satisfaction, and level of daytime fatigue on the VAS scale by choosing a number between 0 and 10. Mean VAS scores for these parameters were compared with other variables in statistical analyses.

### Female Sexual Function Index (FSFI)

The FSFI was developed by Rosen et al. in 2000 to evaluate women's sexual function over the last 4 weeks. The scale is a 19-item multidimensional scale consisting of 6 subscales: desire, arousal, lubrication, orgasm, satisfaction, and pain. Items 3-14 and 15-19 are rated on a 6-point (0-5) Likert-type scale and the other items on a 5-point (1-5) Likert-type scale. Raw scores on this scale range from 4 to 95, and final scores after multiplying by the coefficients range from 2 to 36. The effect coefficients used to calculate the total scale score were determined to be 0.6 for sexual desire, 0.3 for sexual arousal and lubrication, and 0.4 for orgasm, satisfaction, and pain^28^. In a study conducted to determine the cut-off value of the scale, a score of 26.55 was accepted as indicating a decline in sexual function^29^. Therefore, in this study we dichotomized results for the women's sexual function according to this cut-off value. Women who scored >26.55 on the scale were considered to have healthy sexual function, while those who scored ≤26.55 were classified as having sexual dysfunction. The Turkish reliability and validity study of the FSFI was conducted by Aygin and Aslan in 2005^30^. In the validity and reliability study conducted in Turkey, the Cronbach's α of the scale was 0.98 29. In this study, the Cronbach's α coefficient was found to be 0.94.

### Sexual Quality of Life-Female (SQOL-F) Questionnaire

The SQOL-F was developed by Symonds et al. in 2005 to evaluate SQOL in women^31^. The questionnaire consists of 18 items scored on a 6-point Likert type scale. Each item is rated based on the respondent's sex life in the last 4 weeks. The minimum possible score is 18 and the maximum is 108. The scale has no cut-off value, and higher scores indicate better SQOL. The Turkish reliability and validity study of the SQOL-F was conducted in 2005 by Tuğut and Gölbaşı in 2005, who reported a Cronbach's α coefficient of 0.83 32. In the present study, the Cronbach's α coefficient of the scale was 0.95.

### Statistical Analyses

Study data were analysed using SPSS for Windows version 25.0 (IBM Corp, Armonk, NY). Frequency, median, minimum, maximum, mean, and standard deviation were used as descriptive statistics in the data analysis. The Kolmogorov-Smirnov test was used to determine whether the data were normally distributed. Parametric tests were used to analyse scale and subscale scores showing normal distribution. Quantitative variables were analysed using independent samples t test, Mann-Whitney U test, one-way analysis of variance (ANOVA), Kruskal-Walli's analysis, and Bonferroni test for multiple comparisons. Chi-square analysis was performed in independent samples to assess the relationship of qualitative data. Relationships between variables were analysed using correlation analysis. Multiple linear regression analyses were performed to determine the effect of independent variables on the dependent variable. Factors affecting FSFI scores were identified using logistic regression analysis. The logistic regression model was created using factors reported to be associated with SD in the literature and the variables that showed significant differences related to FSFI score in the statistical comparisons made in this study. Although independent-samples t test analysis showed FSFI scores were associated with number of pregnancies, births, and children, only the pregnancy variable was included in the model due to the high correlation between these three variables. Continuous variables in the model were analysed without grouping. The statistical results were analysed within a 95% confidence interval and an alpha value below 0.05 was considered significant.

### Ethics

Before starting the study, ethical approval was obtained from the Eastern Mediterranean University Ethics Committee (ETK00-2021-0094, dated March 23, 2021) and a research permit was obtained from the Ministry of Health. Participants were asked to submit their consent via an online consent form prepared in accordance with the Declaration of Helsinki.

## Results

The women who participated in the study had a mean FSFI total score of 26.91±5.62 (min: 2.60, max: 36.00). In addition, 39.1% (n=252) of the women had an FSFI score of 26.55 or lower, indicating sexual dysfunction. The mean SQOL-F score was 79.08±20.90 (min: 6.67, max: 100).

In this study, comparison of the general characteristics of participants with and without SD according to their FSFI score revealed no statistically significant differences in income level, history of birth, or history of COVID-19. However, there were significant differences between the subgroups in terms of employment status, education level, and partner education level. Moreover, statistical comparisons showed that women with SD were older and had older partners, had more pregnancies, births, and children, had reported lower VAS sexual compatibility and sexual satisfaction scores, higher VAS fatigue level, and lower monthly frequency of sexual intercourse (p<0.05; Table 1).

**Table 1:** Distribution of the participants' general characteristics according to presence of sexual dysfunction

	Total		Sexual dysfunction (n=252)		No sexual dysfunction (n=392)		p Values
Variable	N	%	N	%	N	%	
Employment status							
Working	547	84.9	205	81.3	342	87.2	p=0.043^a^,*
Not working	97	15.1	47	18.7	50	12.8	

Education level							
Elementary school	33	5.1	20	7.9	13	3.3	
High school	80	12.4	36	14.3	44	11.2	p=0.034^a^,*
Associate degree	118	18.3	43	17.1	75	19.1	
Undergraduate or postgraduate	413	64.1	153	60.7	260	66.3

Partner education level							
Elementary school	58	9.0	31	12.3	27	6.9	
High school	122	18.9	48	19.0	74	18.9	p=0.005^a^,*
Associate degree	95	14.8	24	9.5	71	18.1	
Undergraduate or postgraduate	369	57.3	149	59.1	220	56.1	

Income level							
Income exceeds expenses	205	31.8	70	27.8	135	34.4	
Income equal to expenses	340	52.8	141	56.0	199	50.8	p=0.212^a^
Income less than expenses	99	15.4	41	16.3	58	14.8	

Mode of delivery							p=0.338

Number of pregnancies							
	1.62 ±1.26 (0–9)		1.77±1.30		1.53±1.23		p=0.021^b^,*

Number of births							
	1.36 ±1.03 (0–9)		1.51±1.14		1.26±0.94		p=0.002^b^,*

Number of children							
	1.34 ±1.01 (0–9)		1.48±1.12		1.25±0.93		p=0.006^b^,*

VAS sexual compatibility							
	8.03±1.98 (0–10)		6.76±2.18		8.85±1.28		p=0.000^b^,*

VAS sexual satisfaction							
	7.94±2.15 (0–10)		6.60±2.42		8.80±1.39		p=0.000^b^,*

VAS fatigue level	7.01±2.16 (0–10)		7.23±2.35		6.87±2.02		p=0.046^b^,*

Frequency of sexual intercourse per month	7.64±4.96 (1–35)		5.60±3.54		8.95±5.29		p=0.000^b^,*

a Chi-square analysis

b Independent-samples t test

* p<0.05

SD: Standard deviation, VAS: Visual Analog Scale, COVID-19: Novel coronavirus disease 2019.

The results of the logistic regression analysis to identify factors associated with women's SD are shown in Table 2. The model established to demonstrate selected variables' relationship with FSFI score showed statistical significance (X2=272.209; p<0.05). R2, which shows the explanatory power of the model, was between 34.5% and 46.7%. Employment status, partner education, VAS sexual compatibility score, VAS sexual satisfaction score, VAS fatigue level, and monthly frequency of sexual intercourse were significantly associated with FSFI score (p<0.05). Women who were not working were approximately half as likely to have healthy SF compared to those who were working. Women whose partners had associate degrees were 5.462 times more likely to have healthy SF compared to those whose partners had an elementary education level. VAS sexual compatibility score, VAS sexual satisfaction score, and average monthly frequency of sexual intercourse also showed a positive association with healthy SF. In contrast, VAS fatigue score was negatively associated with SF. The model predicted SF or dysfunction according to FSFI score with 80.9% accuracy.

**Table 2:** Logistic Regression Analysis of Factors Associated with Sexual Dysfunction

	β	p	OR (95% CI) *
Employment status (reference: working)			
Not working	-0.661	0.049	0.516 (0.268–0.996)
Education level (reference: elementary school)			
High school	0.321	0.586	1.379 (0.435–4.373)
Associate degree	0.405	0.480	1.500 (0.487–4.616)
Undergraduate or postgraduate	0.548	0.350	1.730 (0.548–5.462)
Partner education level (reference: elementary school)			
High school	0.348	0.425	1.416 (0.602–3.329)
Associate degree	1.698	0.001	5.462 (2.050–14.552)
Undergraduate or postgraduate	0.448	0.303	1.565 (0.667–3.671)
Age	-0.023	0.206	0.978 (0.944–1.013)
Partner age	-0.050	2.330	0.951 (0.902–1.015)
Number of pregnancies	0.116	0.257	1.122 (0.919–1.371)
VAS sexual compatibility	0.518	0.000	1.679 (1.362–2.069)
VAS sexual satisfaction	0.230	0.014	1.259 (1.048–1.512)
VAS fatigue level	-0.120	0.015	0.887 (0.806–0.977)
Frequency of sexual intercourse per month	0.160	0.000	1.173 (1.109–1.242)
Constant	-6.108	0.000	0.002

COX-Snell R2=0.345     Nagelkerke R2=0.467     **X**^2^ (p) =272.209 (0.000)

Overall classification percentage=80.9

* OR: Odds ratio and 95% confidence interval, VAS: Visual analog scale

The results of our multiple regression analysis to identify factors associated with women's SQOL are shown in Table 3. The model showed statistical significance (p<0.05; F=74.666). The variance inflation factor, which describes the relationship between independent variables, was found to be less than 5, indicating no multicollinearity problem in the model. The Durbin-Watson test value was sufficient and no autocorrelation problem was detected. Independent variables explained 63% of the change in SQOL-F score (adjusted R2=0.632). The model indicated that sexual desire (β=2.625), satisfaction (β=1.338), pain or discomfort (β=1.274), age (β=-0.356), VAS sexual compatibility (β=1.984), VAS sexual satisfaction (β=2.068), and VAS fatigue level (β=-0.981) were statistically significantly associated with SQOL-F (p<0.05). Other independent variables were not significantly associated with SQOL (p>0.05).

**Table 3:** Multiple regression analysis of factors associated with sexual quality of life

Variable	*β*	*t*	*p*	Beta	VIF	*F*	*p*	Adj. *R*^2^
Constant	7.238	1.388	0.166					
FSFI sexual desire	2.625	3.381	0.000*	0.127	2.081			
FSFI arousal	1.338	1.553	0.121	0.073	3.839			
FSFI lubrication	0.854	1.128	0.260	0.043	2.587			
FSFI orgasm	-0.057	-0.091	0.928	-0.004	2.823			
FSFI satisfaction	4.114	6.652	0.000*	0.261	2.691			
FSFI pain or discomfort	1.221	2.825	0.005*	0.079	1.376			
Mode of delivery (none)	1.221	0.751	0.453	0.025	1.877			
Age	-0.356	-2.192	0.029*	-0.117	4.961	74.666	0.000	0.632
Partner age	0.271	1.948	0.052	0.101	4.731			
Marriage duration	0.105	0.950	0.342	0.038	2.722			
Number of pregnancies	0.597	1.076	0.282	0.036	1.966			
VAS sexual compatibility	1.984	3.731	0.000*	0.188	4.422			
VAS sexual satisfaction	2.068	4.140	0.000*	0.212	4.602			
VAS fatigue level	-0.981	-4.156	0.000*	-0.101	1.042			
Frequency of sexual intercourse per month	0.148	1.277	0.202	0.035	1.329			

Mode of delivery: No births=1, Durbin-Watson=2.074, VIF: Variance inflation factor, FSFI: Female sexual function index, VAS: Visual analog scale

Correlation analysis revealed significant positive correlations between SQOL-F and FSFI total and sexual desire, arousal, lubrication, orgasm, satisfaction subscale scores. Similarly, there were statistically significant positive correlations between SQOL-F and average monthly frequency of sexual intercourse and VAS levels of sexual compatibility and sexual satisfaction. In contrast, significant negative correlations were detected between SQOL-F and pain or discomfort subscale scores, age, partner age, marriage duration, numbers of pregnancies, births, and children, and VAS fatigue level (Table 4).

**Table 4:** Relationship between SQOL-F and FSFI subscales, VAS scores, and other variables

Variable	SQOL-F	

	r	p
FSFI sexual desire^a^	0.529**	0.000
FSFI arousal^a^	0.643**	0.000
FSFI lubrication^b^	0.546**	0.000
FSFI orgasm^a^	0.568**	0.000
FSFI satisfaction^a^	0.687**	0.000
FSFI pain or discomfort^a^	-0.342**	0.000
FSFI total	0.707**	0.000
Age^a^	-0.157**	0.000
Partner age^a^	-0.111**	0.005
Marriage duration^a^	-0.096*	0.015
Number of pregnancies a	-0.088*	0.026
Number of births^b^	-0.121**	0.000
Number of children^b^	-0.118**	0.000
VAS sexual compatibility^a^	0.674**	0.000
VAS sexual satisfaction^a^	0.678**	0.000
VAS fatigue level^a^	-0.121**	0.000
Frequency of sexual intercourse per month^b^	0.432**	0.000

** p<0.01

* p<0.05

a Pearson correlation test

b Spearman correlation test

SQOL-F: Sexual quality of life–Female questionnaire, FSFI: Female sexual function index, VAS: Visual analog scale

## Discussion

### Sexual function

Sexuality, considered a necessity for continuation of the species on one hand and a basic human need on the other, is an extremely complex and delicate phenomenon that encompasses physical, mental, learning, and social dimensions^33^. The current COVID-19 pandemic has had profound impacts on people's health and routine lives^34^. Based on this, we conducted this study to examine whether the COVID-19 pandemic has affected the SF and SQOL of women living in Turkey.

Studies conducted in different countries of the world have demonstrated a decrease in FSFI scores and an increase in the prevalence of female SD compared to the pre-pandemic period^11^,^35^–^39^. In a study conducted with Turkish couples, the proportion of women scoring 26.55 or lower on the FSFI was 45.4% before the pandemic and increased to 52.6% during the pandemic^22^. The mean FSFI score of the women in the present study was 26.91 ± 5.62, with 39.1% of the women having an FSFI score of 26.55 or lower. In a meta-analysis study published in Turkey shortly before the COVID-19 pandemic, the rate of SD among women was reported to be 39.65% 24. Our findings are similar to the results of this pre-pandemic meta-analysis and contradict those of other studies conducted in Turkey and other countries during the pandemic.

The discrepancy between our findings and those reported in other countries may be due to differences in the impact of COVID-19 among countries and to cultural differences. However, we attribute the difference between our results and those reported in other studies conducted in Turkey to the larger sample included in our study. In response to the first research question, less than half of the women participating in the study had SD, and we concluded that SF was not affected by the pandemic.

### Factors associated with sexual function

As female sexuality is not studied as often as male sexuality, both the etiology and pathophysiology of female SD have not been determined in detail, and thus neither have methods for its treatment^41^. Therefore, in the present study, we also investigated factors associated with women's SF as our second research question. Some studies conducted during the pandemic have demonstrated decreases in sexual intercourse frequency, sexual desire, and FSFI scores and increases in the level of stress related to sexual intercourse in women who were not working paid jobs or were unemployed^18^,^35^,^37^. Consistent with the literature, not working was associated with SD and also emerged as a significant predictor of SD in logistic regression analysis.

It is known that working status and fatigue level are associated and that fatigue affects sexuality. Pre-pandemic studies showed that women with symptoms of fatigue had SD and that increased fatigue adversely affected the sexual cycle^42^–^44^. In the present study, fatigue level was higher in women found to have SD compared to those who did not, and the results of logistic regression analysis showed that fatigue was negatively associated with sexual health. This finding is consistent with the literature examples given.

Studies on the effect of education on SF both before and during the COVID-19 pandemic have yielded contradictory results. Schiva et al. (2020) reported that FSFI scores during the pandemic were lower in women with a high education level^39^. In contrast, Güzel and Döndü (2021) determined that women with low education had lower FSFI scores^45^, and Fuches et al. (2020) reported that education was not associated with SF^37^. A pre-pandemic meta-analysis conducted in Turkey revealed no relationship between women's education and SD 24. Although we determined that low education in women was associated with SD in our study, its relationship with SD was found to be insignificant in logistic regression analysis. On the other hand, low partner education level was associated with SD and high partner education was found to be a significant predictor of healthy SF. Kılıç (2019) also reported in their meta-analysis study that higher partner education level was associated with a higher rate of SD in women^46^. As opposed to the contradictory studies in the literature, our results suggest that more educated male partners may positively affect women's SF by being more involved in domestic responsibilities and more sensitive in their sexual lives.

With age, sexual function in women may be affected by hormonal changes, the onset of chronic diseases, and social and psychological factors^39^,^46^. In a study conducted in Turkey during the pandemic, there was no difference in weekly frequency of sexual intercourse according to age group^18^, whereas another study showed a decrease in sexual desire with older age^22^. Of the two meta-analysis studies conducted in Turkey before the pandemic, there was no relationship between age and SD in one while a decrease in the rate of SD with older age was reported in the other^24^,^46^. In the present study, older age of the woman and partner were associated with SD, but woman and partner age were not a significant predictor of SD in logistic regression analysis.

Compatibility between partners is another important element related to SF. Sexual compatibility was reported to be strongly associated with sexual satisfaction^47^. Witting et al. (2008) found that women with SD had lower compatibility with their partners than those without^48^. Bilge et al. (2020) detected a positive relationship between partner compatibility and FSFI scores in their study of nurses^44^. Klapilova et al. (2014) reported that the frequency of penile-vaginal intercourse and orgasm were positively associated with intimacy, satisfaction, and compatibility of both partners^49^. Omar et al. (2021) reported a relationship between sexual dissatisfaction and SD in their study conducted during the COVID-19 35. We also determined in this study that low sexual compatibility and satisfaction levels were associated with SD, while high sexual compatibility and satisfaction levels were shown to be predictors of SF in logistic regression analysis. This suggests the presence of a virtuus cycle. Sexual compatibility and satisfaction contribute to healthy SF, and healthy SF increases satisfaction and compatibility.

Varying results have been reported concerning the relationship between obstetric history and SF during the COVID-19 pandemic. Two studies suggested that number of children was not associated with SF^37^,^45^. Another study demonstrated a decrease in the frequency of sexual intercourse and sexual desire in people with no children18. In contrast, other studies showed a decrease in FSFI scores with higher parity^39^. In this study, high pregnancy, birth, and children's numbers were found to be associated with SD, but number of pregnancies was not a significant predictor of SD in logistic regression analysis. The relationship between these parameters may be attributable to the physical effects of pregnancy and childbirth on women and the increased care burden with more children.

The pandemic has caused changes in all parts of life, including sexual behaviors and habits^18^. The frequency of sexual intercourse is an important parameter related to this. In the literature there is evidence that frequent sexual intercourse reduces the incidence of some physical diseases, improves psychological wellbeing and quality of life, and is associated with greater enjoyment of life^18^,^50^,^51^. Yuksel and Ozgor (2020) determined that pandemic-induced lockdowns increased the frequency of sexual intercourse in women but decreased their FSFI scores compared to before the pandemic^11^. In the present study, the mean monthly frequency of sexual intercourse was found to be 7.64 ± 4.96. In a pre-pandemic study conducted in Turkey, 42% of women reported their frequency of sexual intercourse as twice a week^52^. Compared to their study, we consider the frequency of sexual intercourse among women participating in this study similar to the pre-pandemic national average^52^. In addition, in this study, it was determined that low frequency of sexual intercourse was correlated with SD and was positively associated with SF in logistic regression analysis. Our findings are consistent with the general literature. Therefore, we concluded that frequent sexual intercourse may contribute to healthy SF.

### Sexual quality of life

A quality sex life is described not only in terms of not having diseases and disabilities affecting reproductive and sexual functions, but also in terms of not having feelings that suppress sexuality, such as fear, shame, and guilt; being able to control one's sexual behavior; and deriving satisfaction. In short, SQOL refers to the state of being satisfied with one's sex life^53^.

Sexual quality of life is a subjective experience that may be affected by the COVID-19 pandemic. However, studies have yielded differing results on this subject. While some studies on the subject showed that women's SQOL decreased during the COVID-19 pandemic^16^,^34^,^39^, no changes were observed in some other studies 23,54. In a study conducted in Turkey, Özlü et al. (2021) reported that women had a lower frequency of sexual activity and their SQOL was at a moderate level^23^. However, another Turkish study showed that participants had significantly poorer SQOL compared to 6-12 months earlier^11^. In the present study, the mean SQOL-F score was 79.08±20.90. In response to our third study question, this shows that the women in our study have moderate to high SQOL during the pandemic.

In two studies evaluating SQOL in healthy women in Turkey before the pandemic, the mean SQOL-F scores were 81.09±22.04, and 71.54±20.46 55,56. Our findings and the results of previous studies indicate that the SQOL among the women in our study was similar to that reported in pre-pandemic studies conducted in Turkey and was not affected by the pandemic. We attribute the difference between our findings and those of other studies conducted during the pandemic to the larger sample size in this study. Variability in results among counties may be due to cultural differences and the different degrees of impact of COVID-19 in each country.

### Factors associated with sexual quality of life

In this study we also investigated factors associated with women's SQOL and the results obtained in this section answer our fourth research question. Schiavi et al. (2020) stated that SD was associated with level of sexual satisfaction and quality of life^39^. Peixoto (2021) reported that women with healthy SF had high sexual satisfaction^13^. The expected association between SF status and SQOL was also supported by our findings that SQOL-F scores positively correlated with FSFI general and subscale scores, and that sexual satisfaction, desire, pain and discomfort were important independent variables affecting SQOL. Ultimately, it is natural for physical health to positively impact SQOL.

The results of the study suggest that the effect of age on SQOL varies. A strong positive relationship was found between partner age and SQOL in one study conducted during the pandemic^23^, while no age-related difference in SQOL was reported in another 16. In a study conducted in Egypt, women whose partners were over 35 years of age reported an increase in the level of stress caused by sexual intercourse^35^. In a study conducted before the pandemic, it was stated that SQOL declined with advancing age^57^. In the present study, the ages of both the women and their partners were negatively associated with SQOL and the women's age also affected SQOL-F scores according to our multiple regression analysis, suggesting that SQOL decreases with age. Furthermore, greater fear of COVID-19 infection and related complications in middle-aged and older individuals compared to young people may have indirectly affected SQOL in this age group.

There are studies demonstrating a relationship between the frequency of sexual intercourse and SQOL. In a study conducted before the pandemic, people who had sexual intercourse 3 to 4 times a month had high SQOL^57^. In another study, women who reported that their sex life with their partner was not affected during the pandemic were found to have higher SQOL^23^. In this study, although we observed a strong positive association between sexual intercourse frequency and SQOL, multiple regression analysis revealed no significant relationship between these parameters.

Mutual pleasure and compatibility are important components of sexual satisfaction and develop not from the absence of sexual dysfunction, but as a result of positive sexual experiences 58. Satisfying sexuality promotes physical and mental health and improves quality of life 59. In the present study, SQOL was strongly positively associated with sexual compatibility between partners and satisfaction. In addition, both of these factors were shown to be significantly associated with SQOL in multiple regression analysis. Consistent with the literature, our findings show that sexual compatibility between partners and satisfaction are important predictors of SQOL.

In all cultures, there are customs related to relationships between men and women, including formal marriage. However, marital relationships often differ among societies because they are guided by culturally determined norms. Factors such as family structure, age, education level, economic status, and having children can affect satisfaction, compatibility, and sex life 60,61. Stress is one of the main causes of reduced sexual desire, and the stress that occurs after childbirth is considerable. The birth of a first child in particular brings about changes in family organization. In addition, children are sometimes a barrier to intimacy between partners 61. In our study, we detected significant negative relationships between pregnancy, childbirth, number of children, marriage duration, and SQOL. In this study, SQOL showed a strong positive relationship with number of pregnancies, births, and children, as well as marriage duration, but multiple logistic regression analysis indicated that marriage duration and number of pregnancies were not associated with SQOL. Motherhood is considered one of the most important and highest priority roles for women in Turkey. Culturally, men have a secondary role in pregnancy, childbirth, and child care, with women being supported mostly by other female members of the family. However, social distancing measures during the COVID-19 pandemic deprived most women of this support from their inner circles. Both of these factors may have negatively affected SQOL.

Adequate sleep and rest are also essential for staying healthy and recovering from illness. Quality sleep and rest support SQOL, and a healthy sex life also contributes to quality sleep and rest^62^. For example, in a study conducted with climacteric women, women with high fatigue were found to have low SQOL 63. In this study, we also observed a strong negative relationship between fatigue level and SQOL. Fatigue also emerged as a significant factor associated with SQOL in multiple regression analysis. Consistent with the literature, our findings show that fatigue level is an important predictor of SQOL.

## Study limitations

The main limitation of this study is that the study sample consisted predominantly of working and educated women. Although we invited women from all walks of life to participate in the online survey, we suspect that conservative and low education women may have been hesitant to answer the questionnaire because it included questions about their sexual life. Secondly, when the questionnaires were examined, we noted that those with unrealistic responses (e.g., number of births >20, monthly frequency of sexual intercourse >70) were submitted by participants who reported a low education level and were not working, and these questionnaires were excluded from the study. We attributed this to mistakes made by low-educated women during data entry because they were not accustomed to using online survey forms. For these two reasons, the study sample consisted mainly of working and educated women, which limits the generalizability of our findings.

## Conclusion

Less than half of the women participating in this study had sexual dysfunction, and overall sexual quality of life was moderate to high. These results are similar to those of pre-pandemic studies conducted in Turkey. Therefore, we conclude that the SF and SQOL of the participants were not affected by the pandemic. In addition, we identified several factors associated with the participants' SF and SQOL. Factors related to female SD in this study were not working, older self and partner age, higher number of pregnancies, births, and children, greater fatigue, and lower female and partner education level, frequency of sexual intercourse, sexual compatibility, and satisfaction. Among these factors, not working and high fatigue level were negatively associated with SF, whereas high sexual intercourse frequency, partner education level, sexual compatibility, and satisfaction were positively associated with SF.

Factors found to vary significantly with SQOL included sexual desire, arousal, lubrication, orgasm, satisfaction, pain or discomfort, frequency of sexual intercourse, level of compatibility between partners and sexual satisfaction, level of fatigue, age of the women and her partner, marriage duration, and number of pregnancies, births, and children. Of these factors, high levels of sexual desire, satisfaction, and compatibility between partners were positively associated with SQOL, while older age, sexual pain or discomfort, and high fatigue level were negatively associated with SQOL.

This large study offers insight about SF and SQOL in healthy women during the COVID-19 pandemic. In addition, the results are important in revealing that modifiable factors such as fatigue, frequency of sexual intercourse, compatibility between partners, and sexual satisfaction are correlates of SF and SQOL. These findings should be supported and causal relationships investigated with prospective randomized controlled trials.
